# Descriptive Values and Diagnostic Utility of a 13-Lead Electrocardiographic System in Healthy Cats and Cats with Cardiac or Non-Cardiac Disease

**DOI:** 10.3390/ani16192993

**Published:** 2026-09-23

**Authors:** Ivona Badric, Nora Schreiber, Barbara Contiero, Marco Baron Toaldo

**Affiliations:** 1 Division of Cardiology, Clinic for Small Animal Internal Medicine, Vetsuisse Faculty, University of Zurich, 8057 Zurich, Switzerland; ivona.badric@uzh.ch (I.B.); nora.schreiber@uzh.ch (N.S.); 2 Department of Animal Medicine, Production and Health, University of Padua, 35020 Legnaro, PD, Italy; barbara.contiero@unipd.it

**Keywords:** arrhythmia, cardiomyopathy, feline, precordial leads

## Abstract

Surface electrocardiogram including peripheral limb and precordial leads has shown clinical utility in humans and dogs, but it is rarely employed in cats. This prospective single-center study aimed to evaluate a 13-lead electrocardiographic system including six peripheral limb and seven precordial leads in a group of healthy cats and cats with cardiac and non-cardiac diseases. To obtain 13 leads, after two minutes of simultaneous recording of the first 12 leads, one lead was moved in another position, and two additional minutes were recorded. The aim was to report descriptive values for several electrocardiographic variables from healthy cats and to test the exploratory diagnostic value to identify diseased status. A total of 125 cats with sinus rhythm were enrolled and grouped as healthy (H, n = 40), cardiac disease (CaD, n = 57) or non-cardiac disease without cardiac involvement (NCD, n = 28). Diagnostic work-up included echocardiography for every cat and different additional diagnostic tests based on the clinical need. Cats were mildly restrained in right lateral recumbency, and peripheral limb and precordial leads were positioned according to an adjusted Wilson system. In total 141 electrocardiographic variables were measured by one operator blinded to group allocation. Descriptive values were obtained from the H group. P wave amplitude was generally higher in peripheral limb leads compared to precordial leads. Thirty-five variables differed between H and CaD on pairwise comparison, while no variable differed between H and NCD. On multivariable logistic regression analysis, R wave amplitude in lead II, aVL, and V6, P wave amplitude in V8, and age were useful in identifying cardiac disease in this cohort. The same variables, without age but including R wave amplitude in V8, were also able to discriminate between H cats and cats with hypertrophic cardiomyopathy phenotype. In our population of cats with sinus rhythm, 13-lead electrocardiogram allowed differentiating H cats from those with CaD and with hypertrophic cardiomyopathy but not from cats with NCD. Descriptive values are provided for different variables.

## Simple Summary

The surface electrocardiogram is a key non-invasive tool for arrhythmia identification. In feline practice, electrocardiograms are conventionally limited to a six peripheral limb lead recording, whereas in human and canine cardiology the addition of precordial leads is standard practice. Precordial leads provide a more comprehensive view of cardiac electrical activity and may improve the recognition of subtle cardiac abnormalities. The aim of this prospective study was to apply a 13-lead electrocardiographic system in cats, to report normal values from a healthy population, and to identify electrocardiographic variables that differentiate cats with cardiac and non-cardiac disease from healthy individuals. Some electrocardiographic variables were associated with cardiac disease, and among these, two variables were derived from precordial leads, while the other two were obtained from peripheral limb leads. P wave amplitudes were generally higher in some peripheral limb leads compared to precordial leads.

## 1. Introduction

Surface electrocardiogram (ECG) is a key tool for evaluating patients with arrhythmia. Moreover, it can help identifying cats with underlying cardiac disease and stratify for risk of cardiac death [1]. However, due to the small patient size, possible limited cooperation, and frequent artifacts from shivering and purring, the analysis of small amplitude deflections, like P waves, might sometimes be challenging in cats [2].

Precordial leads are added to the traditional peripheral limb system by placing a variable number of electrodes at different points on the right and left hemithorax. Several precordial lead systems have been used and validated in people and dogs. In humans, precordial leads improve analysis of atrial depolarizations, intraventricular conduction disturbances, and wide QRS complex rhythms [3]. Studies in dogs have applied precordial leads to aid evaluating ventricular arrhythmias and identify intraventricular conduction disturbances [4,5,6]. However, their clinical value in canine patients remains unclear.

Only a small number of studies report the use of precordial leads in cats, with various techniques of electrode application [2,7]. A recently published study tested a nine-lead precordial system in a population of healthy cats [8]. To date, the clinical value of adding precordial leads to the standard limb system in healthy and diseased cats remains to be evaluated.

This study aims to assess the feasibility and clinical utility of a peripheral limb and precordial lead system in a population of healthy cats and cats with cardiac and non-cardiac diseases. The healthy cats are used to generate normal descriptive values and compared to diseased cats in order to test the discriminatory value of several ECG variables between groups.

We hypothesized that P wave amplitudes are higher in precordial leads compared to peripheral limb leads. Finally, ECG variables obtained from both systems (peripheral limb and precordial) will allow differentiating between healthy cats and cats with cardiac and non-cardiac diseases.

## 2. Materials and Methods

### 2.1. Study Population

This was a prospective, comparative, and explorative single-center study. Between October 2023 and October 2025, cats presented to the Division of Cardiology of the Vetsuisse Faculty at the University of Zurich for cardiological assessment were included. No sample-size calculation was performed, and the number of animals included was based on an exploratory cohort.

To be included, cats had to be one year of age or older, have a complete echocardiographic examination performed, and show phases of sinus rhythm during a four-minute ECG recording. Cats were excluded if not cooperative or if pleural or pericardial effusion (in a quantity more than traces) was present at the time of ECG recording.

For each cat, the following data were annotated: signalment, age, physical examination and cardiac auscultation findings, disease status, and additional diagnostic findings when applicable, including laboratory and non-cardiac imaging data. Presence and type of cardiac and extra-cardiac disease, presence of congestive heart failure (lung edema, and/or pleural or abdominal effusion), and presence of arterial thromboembolism were also annotated. In cats older than seven years, serum thyroid hormone concentration and systemic blood pressure were additionally assessed. Indirect systolic blood pressure was measured using a Doppler device (MD4, Sonomed Ltd., Warsaw, Poland).

Mild sedation was allowed with oral gabapentin (10–20 mg/kg) and/or intramuscular injection of butorphanol (0.1–0.3 mg/kg) to reduce stress and improve patients’ cooperation during diagnostic procedures and ECG recording. No other sedative was allowed.

Cats were divided into three groups based on clinical and echocardiographic data and additional findings according to the diagnostic requirements. Cats were deemed to be healthy (H group) if no clinical signs were reported and if physical and echocardiographic examinations were normal. Cats older than seven years had also to test negative for hyperthyroidism and had to have normal blood pressure values (<160 mmHg). Presence of a soft heart murmur, in absence of echocardiographic evidence of structural heart disease, was not an exclusion criterion. Cats were allocated to the cardiac disease (CaD) group if echocardiographic exam showed any type of acquired or congenital cardiac abnormality, with or without concomitant extra-cardiac diseases, including hyperthyroidism or systemic arterial hypertension. Finally, cats with a normal echocardiographic examination and presence of any type of extra-cardiac disease were classified in the non-cardiac disease (NCD) group.

### 2.2. Echocardiography

All cats underwent echocardiographic examination performed by a board-certified cardiologist or a cardiology resident in training supervised by a board-certified cardiologist. Echocardiographic examinations were conducted according to the established veterinary cardiology standards [9] using an ultrasound machine equipped with phased array transducers of different frequencies (EPIQ 7 ultrasound system, Philips Healthcare, Horgen, Switzerland). Cardiac measurements were obtained from two-dimensional cine loops from right parasternal and left apical views. From a short-axis view, at the level of the papillary muscles, the following measurements were recorded: end-diastolic interventricular septum thickness (measured using a leading edge-to-trailing edge technique), end-diastolic left ventricular free wall thickness (measured using a leading edge-to-leading edge technique), left ventricular end-diastolic and end-systolic internal diameters (measured using an inner edge-to-inner edge technique) and left ventricular fractional shortening. From a right parasternal short-axis basilar view, the left atrial and aortic internal diameters at end-systole were measured, and their ratio calculated. From a right parasternal long-axis view, the internal diameter of the right atrium and left atrium at end-systole and of the right ventricle and end-diastole (at the tricuspid annulus level) were measured using an inner edge-to-inner edge technique. Left ventricular outflow tract maximal velocity was recorded using a continuous wave Doppler from a left apical five-chambers view. Cardiomyopathy phenotypes were classified as hypertrophic, restrictive and non-specific following published guidelines [10]. Clear left ventricular hypertrophy was defined as the presence of any left ventricular wall segments ≥6 mm at end-diastole [11], while values between 5.5 and 5.9 mm were considered equivocal [12]. Moreover, the presence of systolic anterior motion of the mitral valve in combination with normal left ventricular wall measurements was also considered equivocal for a hypertrophic cardiomyopathy (HCM) phenotype. Cats were considered cardiologically healthy if no echocardiographic abnormalities were identified. Moreover, the following cardiac measurements had to be within normal limits: left atrial and right atrial diameter at end-diastole <16 mm and 13.72 mm, respectively [13,14]; ratio between the left atrial and aortic diameter < 1.5 [15]; right ventricular internal diameters at end-diastole (<6.36) [13]; and left ventricular wall thickness at end-diastole <5.5 mm [12].

All echocardiographic studies were reviewed, and measurements were repeated by one single operator at the end of the patients’ inclusion using dedicated software (TomTec Arena, version TTA2.51.04, TOMTEC Imaging Systems GmbH, Unterschleissheim, Germany).

### 2.3. Electrocardiography

Electrocardiographic examinations were obtained using a digital ECG machine (Cube ECG, Cardioline S.p.A., Caverano, Italy) using the following settings: paper speed, 50 mm/s; sensitivity, 20 mm/mV; low-pass filter, 35 Hz; high-pass filter, 0.05 Hz; notch filter, 50 Hz; sampling frequency, 500 Hz.

All the ECG recordings were performed for a duration of four minutes by one operator (IB or MBT). A 13-lead surface ECG was performed with the cats mildly restrained in right lateral recumbency, with the legs slightly stretched. Alligator electrodes were employed and directly placed on the skin, without clipping the hair and using alcohol as an electrical conductor. Peripheral limb leads (I, II, III, aVR, aVL, aVF) were obtained by placing the electrodes at the level of the radius and ulna. Precordial leads were recorded by positioning the electrodes according to Wilson’s precordial lead system, as modified by Kraus et al. [4] and slightly adjusted for this study, as shown in Figure 1. The V1 electrode was positioned at the fourth right intercostal space parasternally. The V2 electrode was positioned at the fifth left intercostal space at the same height as V1, while the V4 electrode was placed at the same intercostal space as V2 but at the level of the costochondral junction. The V3 electrode was placed equidistant from V2 and V4, and the remaining left precordial electrodes (V5 and V6) were placed dorsally, equidistant from each other. After two minutes of recording, the V1 electrode was repositioned at the fifth left intercostal space, approximately two interelectrode distances from V6, at the same height as the scapulohumeral joint (V8), and the recording was repeated for two additional minutes.

Each ECG trace was analyzed at the end of the study by a single operator (MBT) blinded to the patient’s group allocation. All measurements were performed on printed ECG, using a manual caliper with a precision of 0.025 mV and 10 ms.

Only traces showing at least three consecutive sinus beats were included; measurements were performed on those beats, and the mean was calculated. Moreover, only good-quality ECGs with minimal artifacts and “clean” and straight isoelectric lines were retained. Rhythm diagnoses included sinus rhythm, sinus arrhythmia or pathological rhythms and were based on the following criteria; normal sinus rhythm: normal heart rate coupled to regular sinus P waves, with less than 10% variation in R-R intervals; sinus bradycardia and sinus tachycardia: sinus rhythm with heart rate below or above 140 and 220 beats per minute, respectively; atrial premature complex: a premature narrow QRS complex conducted by a P wave with an abnormal morphology; supraventricular tachycardia: more than three consecutive atrial premature complexes with a heart rate above 220 beats per minute; ventricular premature complex: premature wide and bizarre QRS complex without an associated P wave [16]; accelerated idioventricular rhythm: more than three ventricular premature complexes at a normal heart rate; ventricular tachycardia: more than three ventricular premature complexes at a heart rate above 220 beats per minute [1,17]. Heart rate was calculated by counting the number of QRS complexes included within a three-second interval and multiplied by twenty.

Several ECG variables were measured from all limb and precordial leads, as shown in Figure 2.

Logarithmic formula (QTc=log600×QT/logRR) [18] was used to correct the QT interval in lead II for the heart rate (QTc). Although this equation has not been validated in cats, it showed the greatest accuracy in calculating the canine QTc compared with other methods, and it has also been used in other feline studies [1,18]. The mean electrical axis on the frontal plane for P and T waves and QRS complex was calculated using the following formula MEA=arctan(Iamp,aVFamp)×180/π [19]. 

### 2.4. Statistical Analysis

All continuous variables were assessed for normality using the Shapiro–Wilk test. Body weight among groups was compared using an ANOVA test, while sex distribution was compared with a Fisher’s exact test. All other variables among groups were compared using a non-parametric Kruskal–Wallis test (H vs. CaD vs. NCD). Post hoc multiple pairwise comparisons were performed using the Steel–Dwass–Critchlow–Fligner procedure. To account for the multiplicity arising from the evaluation of 141 ECG variables, the overall Kruskal–Wallis *p*-values were additionally adjusted using the Benjamini–Hochberg false discovery rate procedure. This adjustment was applied only to the study-wide univariate comparisons and was not used as a screening criterion for the multivariate logistic analysis, which was considered a separate exploratory analysis. P wave amplitudes across the 13 leads were compared separately within each study group using the non-parametric Friedman test for related samples. When the global Friedman test was significant, post hoc pairwise comparisons between leads were performed using Conover’s multiple-comparison procedure. Because the Friedman analysis requires complete repeated measurements, cats with a missing P wave amplitude in one or more of the leads were excluded from the corresponding within-group analysis. Data are reported as mean ± standard deviation or median (first–third quartile).

Preliminary univariate logistic analyses were performed to identify the most significant predictors, including age (introduced as a continuous variable) and sedation status. A subsequent collinearity analysis helped to detect collinear predictors, which were excluded from further analyses. Collinearity among predictors was assessed using the variance inflation factor (VIF). VIF values close to 1 indicate little or no multicollinearity, whereas increasing VIF values indicate increasing levels of collinearity. Values > 5 were considered indicative of potentially problematic multicollinearity, while values > 10 were considered indicative of substantial multicollinearity, although these thresholds should be regarded as rules of thumb rather than absolute criteria [20]. Finally, a multivariate logistic regression analysis with forward selection was conducted to identify the most significant predictors. For the continuous predictors retained in the final multivariable model, the assumption of linearity in the logit was assessed by adding a quadratic term for each predictor to the multivariable model, one at a time, after mean-centering the corresponding variable. A statistically significant quadratic term was considered evidence of departure from a linear functional form. Internal validation of the multivariable modelling procedure was performed using 2000 bootstrap resamples. In each bootstrap sample, the entire forward-selection procedure was repeated using the same candidate predictor set and entry criterion (*p* < 0.05). The model selected in each bootstrap sample was then applied to the original dataset. Optimism was estimated as the mean difference between the area under the curve (AUC) obtained in the bootstrap sample and that obtained when the corresponding model was applied to the original dataset. The optimism-corrected AUC was calculated by subtracting the mean optimism from the apparent AUC. Selection frequencies of individual predictors across bootstrap samples were also calculated to assess model-selection stability.

To evaluate the potential influence of sedation on ECG measurements, sedation status (0 = non-sedated; 1 = sedated) was compared with the quantitative ECG variables considered as candidate predictors using point-biserial correlation analysis. *p*-values were adjusted for multiple testing using the Benjamini–Hochberg false discovery rate procedure.

To explore whether cardiac treatment may have influenced the ECG phenotype recorded at enrollment, an additional sensitivity analysis was performed within the CaD group. Cats receiving cardiac medication at the time of ECG recording were compared with untreated CaD cats for the ECG variables retained in the final multivariable model. Because these variables were not assumed to be normally distributed, comparisons were performed using the Mann–Whitney U test.

To evaluate the robustness of the multivariable findings, a sensitivity analysis was performed only considering H cats and cats from the CaD group with HCM and excluding other cardiac diseases and cats with an equivocal diagnosis of HCM.

*p*-values < 0.05 were considered significant. All statistical analyses were performed using SAS version 9.4 (SAS^®^ software, SAS Institute Inc.: Cary, NC, USA; 2013) and MedCalc version 22.016 (MedCalc^®^ Software Ltd., Ostend, Belgium).

## 3. Results

### 3.1. Study Population

A total of 168 client-owned and staff-owned cats were initially enrolled in the study. Cats were presented to the cardiology service for clarification of heart murmur, respiratory complaints, rhythm disturbances, limb lameness, collapse, acute blindness, or systemic clinical signs potentially associated with heart disease (vomiting, hyporexia). Additional indications included clarification of cardiomegaly or free fluids in the thoracic and/or abdominal cavity diagnosed during emergency bedside ultrasound or radiography. Other cats were presented for annual cardiac follow-up, health screening examinations, and cardiological examination for breeding purposes.

Of these, 125 cats met the inclusion criteria and were included in the final analysis: 40 cats in the H group, 57 in the CaD group, and 28 in the NCD group.

Cats in CaD group (9 [1,2,3,4,5,6,7,8,9,10,11,12,13,14,15,16,17,18] years) and NCD group (10.5 [1,2,3,4,5,6,7,8,9,10,11,12,13,14,15,16,17] years) were older than cats in H group (4 [1–15 years]) (*p* = 0.006 and *p* = 0.002, respectively), with no difference between CaD and NCD (*p* > 0.999). Sex distribution and body weight did not differ between groups (*p* = 0.35 and *p* = 0.507, respectively).

In total, 18 breeds were represented. The most common was European shorthair (n = 66, 52.8%), followed by British shorthair (n = 18, 14.4%), Maine coon (n = 10, 8%), Bengal (n = 5, 4%), Norwegian forest cat (n = 4, 3.2%), Devon rex, Siberian cat, and Scottish fold (n = 3 each, 2.4%). Less frequently represented breeds included Persian cat, Ragdoll, Selkirk rex longhair (n = 2 each, 1.6%), Birman, British longhair, Burma, Chartreux, Exotic longhair, Russian blue and Turkish Angora cat (n = 1 each, 0.8%).

Mild sedation was administered to 31/125 (24.8%) cats. Butorphanol was used as single sedative in 21/31 cats (5 in the H group, 14 in the CaD group, and 2 in the NCD group), with a dose ranging from 0.1 to 0.3 mg/kg and given intramuscularly. Nine of thirty-one cats received gabapentin, given 90–120 min prior to examination (three cats in each group), with a dose ranging from 10 to 20 mg/kg, administered orally. Two of these nine cats, one cat in the CaD group and one cat in the NCD group, received butorphanol additionally to gabapentin once in the clinic. One of thirty-one cats received pregabalin (5 mg/kg q12 h) for chronic neuropathic pain.

Of the 57 cats in group CaD, 33 (58%) were diagnosed with HCM phenotype. Of these cats 13 (39%) had SAM. One cat with HCM phenotype was diagnosed with an apical thin and hypokinetic segment. Two cats with an HCM phenotype presented with arterial thromboembolism (3.5%), and four cats with congestive heart failure (7%). In these cats, ECG was recorded once the animals were stabilized for their adverse event. Fourteen of fifty-seven (25%) cats were classified as equivocal for HCM. Of these, 8/14 (57%) had an equivocal left ventricular wall thickness, 5/14 (36%) had an equivocal left ventricular wall thickness and SAM, while 1/14 (7%) had SAM only. A non-specific cardiomyopathy phenotype was diagnosed in 6/57 cats (10.5%). Restrictive cardiomyopathy phenotype was diagnosed in 2/57 cats (3.5%). Mitral valve stenosis and subaortic stenosis were each diagnosed in 1/57 (1.7%).

In the CaD group, 27/57 cats (47%) had a concomitant systemic disease. Systemic diseases included hyperthyroidism (n = 10, 37%), chronic kidney disease (n = 6, 22.2%), systemic hypertension (n = 6, 22.2%), chronic enteropathy (n = 3, 11.1%), acute gastroenteritis, adrenal gland tumor, anemia, asthma, lung pathology of unknown origin (n = 2 each, 7.4%), cystolithiasis, diabetes mellitus, feline lower urinary tract disease, lymphadenomegaly, abdominal mass, dental complaints, hepatopathy, pancreatitis, hyperaldosteronism, and vitamin B deficiency (n = 1 each, 3.7%). Some cats had more than one disease at the same time. In the CaD group, 29 cats (50.9%) were receiving treatment for their cardiac disease at the time of inclusion. Medications included clopidogrel (18/29 cats), furosemide (8/29 cats), rivaroxaban (5/29 cats), atenolol (4/29 cats), amlodipine (3/29 cats), torasemide (3/29 cats), benazepril (2/29 cats), sotalol (2/29 cats), and telmisartan (2/29 cats).

Chronic kidney disease was the most common diagnosis in the NCD group together with chronic enteropathy and hyperthyroidism (n = 4 each, 14.3%). Acute gastroenteritis, chronic bronchial disease, and lung masses were found in two cats each (7.1%). Other diagnoses included anemia, cystolithiasis, diabetes mellitus, ear canal cyst, epilepsy, feline infectious peritonitis, feline lower urinary tract disease, fibrosarcoma, humerus fracture, hypokalemia post thyroidectomy, idiopathic head tremor, liver carcinoma, lymphadenomegaly of sternal lymph nodes, a mass in the cranial mediastinum, neuropathic pain, otitis, pyelonephritis, pyoderma, retinal ulcer due to *Mycoplasma felis* infection, retrobulbar lesion, small cell lymphoma, squamous cell carcinoma, systemic hypertension and uveitis (n = 1 each; 3.6%). Some cats had ore that one disease at the same time.

Echocardiographic variables and results of the pairwise comparison are summarized in Appendix A, Table A1.

### 3.2. Electrocardiographic Findings

In the H group (n = 40), sinus arrhythmia was identified in 2/40 cats (5.0%), one of which also presented a single ventricular escape beat during the recording. Isolated ectopy comprised a single ventricular premature complex in 1/40 (2.5%), and atrial parasystole in 1/40 (2.5%). A run of non-sustained accelerated idioventricular rhythm was recorded in 1/40 (2.5%). This cat had a concurrent sinus bradycardia.

In the CaD group (n = 57), ventricular premature complexes were recorded in 7/57 cats (12.3%), and atrial premature complexes in 4/57 cats (7.0%). Sinus tachycardia was identified in 11/57 cats (19%).

In the NCD group (n = 28), sinus arrhythmia was the most common finding, identified in 6/28 cats (21.4%). One of these cats also showed a single ventricular premature complex. Sinus bradycardia was observed in 5/28 cats (17.9%), four of which had concurrent sinus arrhythmia.

Descriptive values for each variable for all 13 leads were obtained from the H group (Table 1).

Pairwise comparison identified 35/141 ECG variables as different between H and CaD groups, while no variable differed between the NCD and H and CaD groups (Table 1). The Benjamini–Hochberg false discovery rate correction was applied to all 141 overall Kruskal–Wallis tests (Appendix A, Table A2). Eleven variables remained significant after adjustment. The false discovery rate adjustment was not used as a screening criterion for the multivariate logistic analysis, which was considered a separate exploratory analysis aimed at identifying independent predictors of cardiac disease. Age and sedation were also considered in the univariate analysis, but only age remained statistically significant (*p* = 0.002).

Following the assessment of multicollinearity, 27 ECG variables were retained as candidate predictors for the multivariable logistic regression analysis. Age was additionally included as a continuous candidate covariate because of its potential confounding effect, resulting in a total of 28 candidate predictors. Due to missing values across the candidate predictors, the multivariable analysis was performed on 94 complete cases, comprising 55 cats with CaD and 39 H cats. Forward multivariable logistic regression selected R wave amplitude in lead II, aVL, and V6, P wave amplitude in V8, and age. A 0.05 mV increase in R wave amplitude in lead II was associated with an odds ratio (OR) of 1.51 (95% confidence interval [CI], 1.21–1.88); a 0.05 mV increase in R wave amplitude in aVL with an OR of 1.54 (95% CI, 1.10–2.15); a 0.05 mV increase in R wave amplitude in V6 with an OR of 1.18 (95% CI, 0.98–1.41); and a 0.05 mV increase in P wave amplitude in V8 with an OR of 2.54 (95% CI, 1.12–5.74). Age was retained by the selection procedure, with an OR of 1.14 per one-year increase (95% CI, 1.00–1.30) (Table 2). In the final model, R wave amplitude in lead II and aVL and P wave amplitude in V8 remained significant, whereas R wave amplitude in V6 and age showed borderline associations (*p* = 0.073 and *p* = 0.053, respectively). The latter two variables had met the predefined significance criterion at the step at which they entered the forward-selection procedure (*p* < 0.05), but their statistical significance was attenuated after inclusion of the subsequent predictors in the final model. No evidence of departure from linearity in the logit was detected for any of the continuous predictors retained in the final multivariable model (all quadratic terms *p* > 0.05). The model derived from the original dataset had an apparent AUC of 0.884. Internal validation based on 2000 bootstrap resamples, with repetition of the entire forward-selection procedure within each resample, yielded a mean optimism of 0.09 and an optimism-corrected AUC of 0.795. Bootstrap selection frequencies showed that R wave amplitude in lead II was selected in 67.5% of resamples, followed by P wave amplitude in V8 (51.8%), R wave amplitude in aVL (42.3%), age (37.2%), and R wave amplitude in V6 (15.7%). These findings indicated greater stability for some predictors than for others.

Sedation status showed only weak associations with the ECG variables considered. At the nominal significance level, P wave amplitude in aVR (r = −0.236, *p* = 0.022), in aVF (r = 0.219, *p* = 0.034), and in V4 (r =0.286, *p* = 0.005) was associated with sedation status. However, none of these associations remained significant after Benjamini–Hochberg false discovery rate correction across the 27 comparisons (all false discovery rate-adjusted *p* > 0.05). Moreover, none of these three ECG variables was retained in the final multivariable model. Overall, these findings provided no evidence of a robust association between sedation status and the ECG measurements evaluated.

A sensitivity analysis was performed by restricting the cardiac disease group to cats with a definite HCM phenotype, resulting in a more homogeneous population of 72 cats (39 H and 33 HCM). Forward multivariable logistic regression selected R wave amplitude in V8, R wave amplitude in lead II, R wave amplitude in V6, R wave amplitude in aVL, and P wave amplitude in V8 (Appendix A, Table A3). In the final model, P wave amplitude in V8 (*p* = 0.012), R wave amplitude in lead II (*p* < 0.001), and R wave amplitude in aVL (*p* = 0.003) remained significantly associated with HCM, whereas R wave amplitude in V6 showed a borderline association (*p* = 0.065), and R wave amplitude in V8 was not significant (*p* = 0.551). The model was globally significant (likelihood-ratio *p* < 0.0001) and showed high apparent discrimination (AUC = 0.944). Notably, four of the five variables selected in the primary H versus CaD analysis were also selected in the HCM-restricted analysis (R wave amplitude in lead II, aVL, and V6 and P wave amplitude in V8), with three of them retaining statistical significance in the final sensitivity model.

At enrollment, 29 of 57 cats in the CaD group were receiving cardiac medication, whereas 28 were untreated. In a sensitivity analysis restricted to CaD cats, none of the ECG variables retained in the final multivariable model differed significantly between treated and untreated cats. These findings did not provide evidence of a major association between treatment status and the principal ECG variables identified by the multivariable analysis.

P wave amplitude differed significantly in nearly all pairwise comparisons across all 13 leads in each group (Table 3, Table 4 and Table 5; Figure 3). Peripheral limb leads consistently showed higher P wave amplitudes compared to precordial leads. However, P wave amplitude in lead I did not usually differ from precordial leads in group H and NCD (*p* = 0.398–0.991), and it usually showed lower values compared to leads V5–V6 in the CaD group.

R/S ratios were assessed in precordial leads among the three groups (Appendix A, Table A4). In lead V1, the R/S ratio was predominantly <1 in all groups (57.5% in H group, 69.1% in CaD group, 62.5% in NCD group). In the left precordial leads (V2–V8), R/S was predominantly >1 in all groups.

## 4. Discussion

A 13-lead ECG system in cats has a moderate accuracy in differentiating between H cats and cats with CaD but not with NCD. Contrary to our initial hypothesis, however, absolute P wave amplitudes in peripheral limb leads were higher than in precordial leads, while among precordial leads, P wave amplitude in lead V1 had the lowest absolute value. Adding lead V8 likely does not improve the detection of sinus P waves, since no difference with other left precordial leads was observed in any of the three groups.

In humans, standard 12-lead ECG includes six unipolar precordial leads (V1–V6) distributed across the anterior and lateral walls of the chest [3]. V1 and V2 electrodes are placed on the fourth intercostal space at the right and left sternal border, respectively, V3 midway between V2 and V4, and V4 on the fifth intercostal space at the midclavicular line. V5 and V6 are positioned on the horizontal plane of V4, at the anterior axillary and midaxillary lines. Additional posterior leads (V7–V9) have been introduced to improve detection of electrical activity arising from regions of the left ventricle that are poorly represented by the conventional six precordial leads, in particular the inferobasal and posterolateral walls. Among them, V8 is placed over the tip of the scapula on the same horizontal plane as V6 [3]. In humans, V7–V9 increase the spatial coverage of the chest, particularly over the inferobasal and posterolateral left ventricular wall [21,22]. By analogy, the V8 position adopted in our protocol was intended to extend the coverage of the feline chest beyond the conventional six precordial leads and to optimize the recording angle for atrial signals projected toward the left dorsolateral thorax. Therefore, the V8 electrode was placed at the fifth left intercostal space at the level of the scapulohumeral joint.

Several publications have established reference values for feline peripheral limb leads and described the influence of body position on the recorded waveform [23,24,25]. A recent study [8] investigated the utility of selected precordial leads (V1, V2 and V4) in healthy cats and, similarly to our study, the authors observed lower P wave amplitudes in precordial leads, with the lowest values recorded in V1. In dogs, some precordial lead systems have also been used to better identify P waves. In one study, Santilli et al. [5] showed that relocating V1 to the right first intercostal space yielded more reliable detection of right atrial activity across different thoracic conformations. Kurogochi et al. [26] tested an extended precordial system including the posterior lead V10 and two bipolar leads (M6M1 and M6M2). On those leads, P wave amplitudes appeared higher compared to the conventional limb and precordial leads, similarly to the Lewis lead system employed in human patients [27]. In humans, the synthesized 18-lead ECG extends the standard system with the back leads V7–V9, and the P wave morphology in V7 has been shown to differentiate atrial arrhythmias arising from anatomically adjacent structures [28]. Based on our results, however, it appears that the electrical vector of sinus P waves in cats does not align with this direction; therefore, in cats, left precordial leads with or without cardiac disease do not improve the detection of these low-voltage events.

Among the ECG variables evaluated in the H group, QRS complex duration differed from reference values previously reported in the literature. Indeed, whereas an upper limit of normality of <40 ms is generally reported for healthy cats [2,16,19], the third quartile of QRS duration in lead II was 60 ms in our cohort of 40 healthy cats. Although our control population was not large, it was sufficiently sized to provide reliable descriptive values. Furthermore, all cats underwent echocardiographic examination to confirm the absence of structural cardiac disease, and all ECG measurements were performed by an experienced cardiologist who was blinded to the animals’ clinical status. One possible explanation for the longer QRS durations observed is the improved quality of contemporary digital ECG systems. Higher signal resolution, together with the recording settings used, may have facilitated the identification of low-amplitude deflections, particularly the Q and S waves, thereby resulting in slightly longer measured QRS durations compared with those reported in earlier studies. Nevertheless, the possibility that a small number of cats had subclinical intraventricular conduction disturbances cannot be completely excluded, and this may have contributed to a mild prolongation of QRS duration in our cohort. Future studies should consider that high-resolution ECG recordings in cats may yield longer measured QRS durations than those previously reported. This should be taken into account when interpreting QRS abnormalities, including wide QRS complex rhythms and bundle branch blocks.

Multiple ECG variables differed between the H and CaD groups, whereas none was able to discriminate between H and NCD cats. Of the 35 variables that differed between H and CaD on pairwise comparison, four ECG variables and age were retained as predictors of CaD in the multivariable model. These were R wave amplitude in lead II, R wave amplitude in lead aVL, R wave amplitude in V6, and P wave amplitude in V8. However, age and R wave amplitude in V6 were borderline significant and therefore likely less relevant; they were not retained as significant variables when a sub-analysis including only cats with the HCM phenotype was performed. In a recent study [29], R wave amplitude in lead II showed the highest discriminatory value between healthy cats and cats with compensated (pre-clinical) HCM, using a cut-off value of >0.26 mV. However, in the study of Gardner et al. [29], the accuracy of this variable was relatively low, since all cats in the diseased group were in the subclinical stage of disease. In our study, a 0.05 mV increase in R wave amplitude in lead II was associated with 1.54-fold higher odds of having CaD and 1.9-fold higher odds of having an HCM phenotype. In humans, increased R wave amplitude in aVL is recognized as a component of the Cornell voltage criteria for left ventricular hypertrophy [30] and has been associated with increased cardiovascular morbidity and mortality in the general population [31]. A similar interpretation may apply to feline structural heart disease, in which a thickened lateral left ventricular wall could shift voltage toward aVL. Although our study was not restricted to cats with left ventricular hypertrophy, HCM was the most prevalent type of cardiac abnormality, accounting for 33 of the 57 patients in the group, while 14 cats were equivocal for HCM. Indeed, maximal left ventricular wall thickness showed higher values in the CaD group as compared with the other two groups. Moreover, R wave amplitude in aVL remained significant also when only HCM cats were considered for the sub-analysis. Therefore, it is possible that increased R wave amplitude in aVL predicts left ventricular hypertrophy in cats as in humans. Similarly, R wave amplitude in precordial leads, V6 and V8, were borderline significant in predicting CaD and HCM. This supports the evidence that R wave amplitudes likely reflect the presence of left ventricular wall hypertrophy, as observed in other studies [29].

The P wave amplitude in V8 was identified as another predictor for CaD on multivariable analysis. We hypothesize that this reflects the high prevalence of left atrial enlargement in feline cardiomyopathy [14] and supports the contribution of left precordial leads to the assessment of atrial activity. This observation is in line with the findings of Santilli et al. [8], who reported higher P wave amplitudes in left than in right precordial leads in healthy cats. Since cats in the H group were younger than diseased cats, age was analyzed as a continuous variable in the multivariable analysis, and it resulted as a risk factor for CaD and HCM, along with the other already-mentioned ECG variables. This finding has been reported in previous studies, showing that older cats have a higher risk of developing HCM and highlighting the possible age-related increase in expression of an abnormal phenotype, as in people [32].

QT and QTc intervals did not differ significantly between the H and CaD groups in our population. This contrasts with the findings of Romito et al. [1], who reported prolongation of QT and QTc on a six-lead ECG in cats with left ventricular hypertrophy. Similarly, Bastos et al. [33] documented longer QT intervals and increased QT instability in cats with an HCM phenotype compared with healthy controls. Possible reasons for this discrepancy include the heterogeneity of our CaD group. Most cats in the CaD group had cardiomyopathy, with several phenotypes represented, while a smaller proportion presented with valvular or other cardiac diseases. In addition, cats were enrolled at variable disease stages. By contrast, Romito et al. [1] and Bastos et al. [33] specifically enrolled cats with a hypertrophic phenotype.

Our findings on the precordial R/S ratio were comparable to those described by Santilli et al. [8]. The R/S ratio was predominantly <1 in V1 across groups, while in left precordial leads (V3–V8) the R/S ratio was >1 in most cats. The predominant R/S patterns were detected among groups; the trend toward higher R/S ratios in V3–V8 in CaD is consistent with a leftward and dorsal shift of the QRS vector in the presence of left ventricular hypertrophy [19].

Several limitations should be acknowledged. First, the groups including diseased cats showed a variety of different cardiac and non-cardiac diseases. Moreover, some cats in the CaD group also suffered from non-cardiac and systemic diseases. However, we believe that this is more representative of the real-life conditions typical of daily practice. The intent was mainly to evaluate the discriminatory value of ECG between healthy and diseased cats and to assess if non-cardiac diseases might affect electrocardiographic variables. To overcome this limitation, we ran a sub-analysis comparing only H cats and cats from the CaD group that showed an HCM phenotype. The results were similar to those of the original analysis, retaining as significant predictors of disease almost all the same variables (namely R wave amplitude in leads II, aVL, V6, and V8 and P wave amplitude in V8).

Second, mild sedation was allowed for some cats, to reduce stress and improve the quality of the examination. Recent randomized studies in cats have shown that a single oral dose of gabapentin produces only minor changes in cardiovascular parameters [34,35] and does not significantly affect standard ECG variables [36]. Butorphanol was the only opioid sedative used in our study, while alfaxalone was not allowed. In one study of healthy cats, combined intramuscular administration of alfaxalone and butorphanol did not produce clinically relevant changes in echocardiographic measurements, but no effect on the ECG was assessed [37]. To our knowledge, no study has specifically evaluated the effect of butorphanol alone, or the combination of butorphanol with gabapentin, on ECG variables in cats. Our analysis showed that no association was present between sedation status and any ECG variable.

Some cats in the CaD group were receiving medications, such as diuretics and antiarrhythmics, that might affect some ECG variables, such as amplitude or heart rate. Although the principal ECG variables identified by the multivariable analysis did not differ between treated and untreated CaD cats in the sensitivity analysis, a treatment effect cannot be excluded. Treatment was not randomly assigned and was prescribed according to clinical indication; therefore, differences between treated and untreated cats may also reflect disease severity and other clinical characteristics. Consequently, the present study cannot fully disentangle disease-related ECG changes from treatment-related effects.

Third, although electrodes were placed by only two operators, following a standardized protocol, some small inaccuracies in limb or precordial lead position cannot be excluded. Anxious cats in which the forelimbs might not have been optimally extended, and overweight cats in which the intercostal spaces and sternum could be difficult to palpate, may have had incorrect electrode positioning. Additionally, to record 13 leads, the V1 electrode was replaced in the V8 position. Therefore, a simultaneous recording of all 13 leads was not possible. Slight changes in heart rate or stress conditions during the two phases of recording might have influenced the results.

Fourth, the population was recruited at a single referral cardiology service with one ECG machine and a specific setting and is therefore not necessarily representative of the general feline population. In particular, the sampling frequency of 500 Hz, while widely adopted across the literature, may have been insufficient to accurately resolve ECG waveforms in cats, whose characteristically elevated heart rates demand greater temporal resolution.

Fifth, this study included only cats in sinus rhythm. It was not our aim to assess the accuracy of ECG in diagnosis any specific arrhythmia nor the adjunct value of precordial leads in identifying non-sinus P waves. It is indeed possible that in some cases, when P’ waves are not visible in peripheral limb leads, precordial leads might help in visualizing these low voltage events, especially if the P’ wave axis is significantly deviated, like in the case of atrial tachycardia or retrograde P’ waves during bypass mediated or junctional rhythms.

Finally, regarding the multivariable model, the apparent discrimination of the model derived from the original dataset was high (AUC = 0.884). However, because predictor selection and model estimation were performed in the same cohort, this value is expected to overestimate the performance. Internal bootstrap validation was therefore performed by repeating the entire variable-selection procedure in each of 2000 bootstrap samples. After correction for optimism, the AUC decreased to 0.795. Thus, although part of the apparent performance was attributable to model development in the same dataset, the modelling procedure retained acceptable-to-good discriminative ability after internal validation. Bootstrap selection frequencies also showed that the composition of the model was not completely stable. R wave amplitude in lead II showed the greatest stability and was selected in approximately two-thirds of bootstrap samples, whereas P wave amplitude in V8 was retained in approximately half. The remaining variables were selected less consistently, particularly R wave amplitude in V6. This variability probably reflects the relatively limited sample size and the presence of several ECG measurements providing partially overlapping information. Consequently, the individual variables retained in the final model should not be regarded as definitive predictors of cardiac disease but rather as variables associated with cardiac disease in this cohort whose relevance requires confirmation in independent populations. The sensitivity analysis restricted to cats with a definite HCM phenotype provided additional support for the primary multivariable findings. Despite restricting the analysis to a smaller but clinically more homogeneous cardiac population, four of the five variables selected in the primary analysis were retained by the forward-selection procedure. In particular, R wave amplitude in lead II, aVL, and V6 and P wave amplitude in V8 remained significantly associated with disease status in the final HCM-restricted model, with R wave amplitude in V8 as an additional significant variable. This consistency suggests that the associations observed for these ECG measurements were not solely driven by the heterogeneity of cardiac abnormality included in the overall CaD group.

## 5. Conclusions

In conclusion, a 13-lead ECG system including peripheral limb leads and seven precordial leads can be applied in cats. Peripheral limb leads have the highest P wave amplitudes among all leads. V8, however, provides higher P wave amplitudes than V1 and may assist in the assessment of atrial electrical activity. The present study also provides descriptive values from healthy adult cats for peripheral limb leads and, as a novel contribution, for seven precordial leads. Some precordial and peripheral limb lead variables, in particular R wave amplitude in lead II and aVL and P wave amplitude in V8, were predictors of CaD, while no ECG parameter differed between cats with NCD and H cats. Moreover, the same variables were useful in differentiating between H cats and cats with an HCM phenotype.

## Figures and Tables

**Figure 1:**
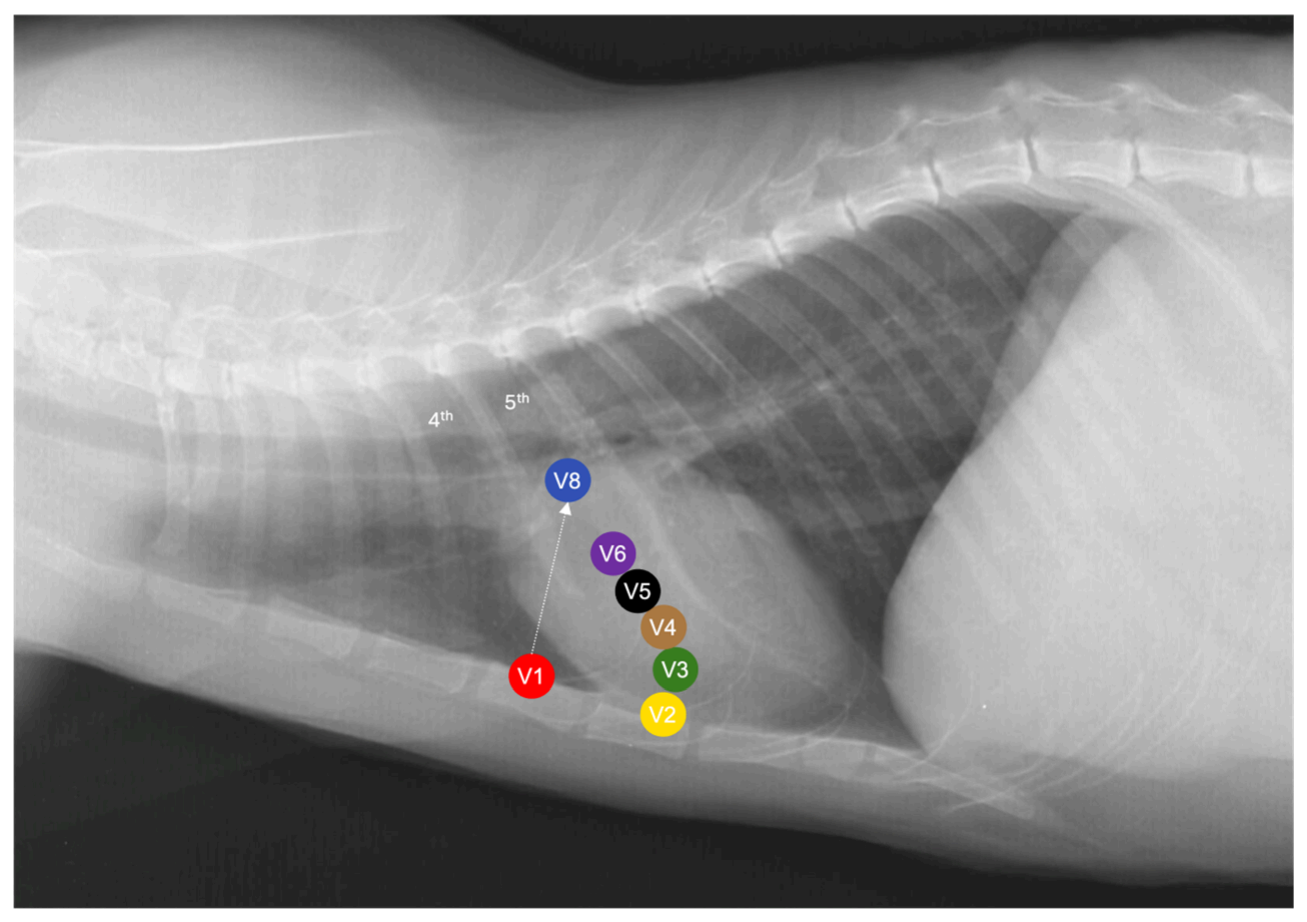
Thoracic radiograph of a cat showing the site of application of the electrodes to obtain seven precordial leads. After the first electrocardiographic recording, the V1 electrode was moved from the fourth right intercostal space (4th) to the fifth left intercostal space (5th), approximately at the level of the scapulohumeral joint. All left electrodes are placed equidistant, with a double distance between V6 and V8.

**Figure 2:**
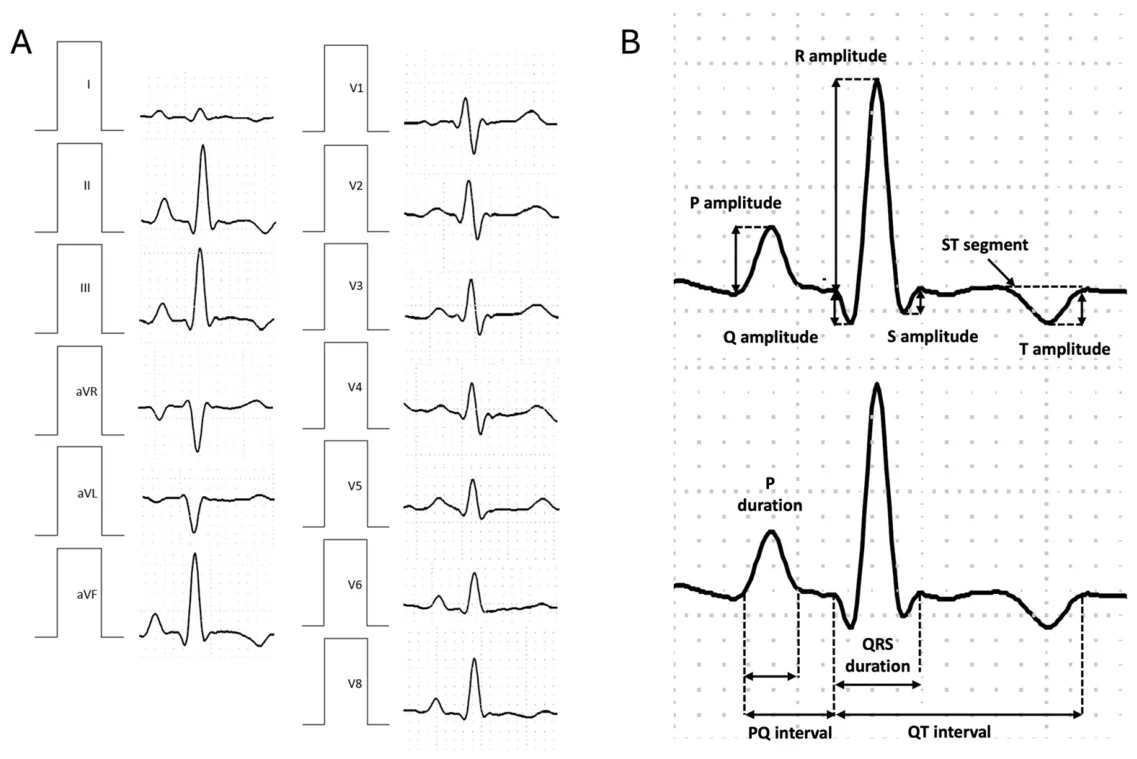
(**A**): Composite image showing a normal sinus beat from all thirteen leads, obtained from a cat in the study. (**B**): Zoomed lead II complex, showing all the measurements of waves, complexes and intervals.

**Figure 3:**
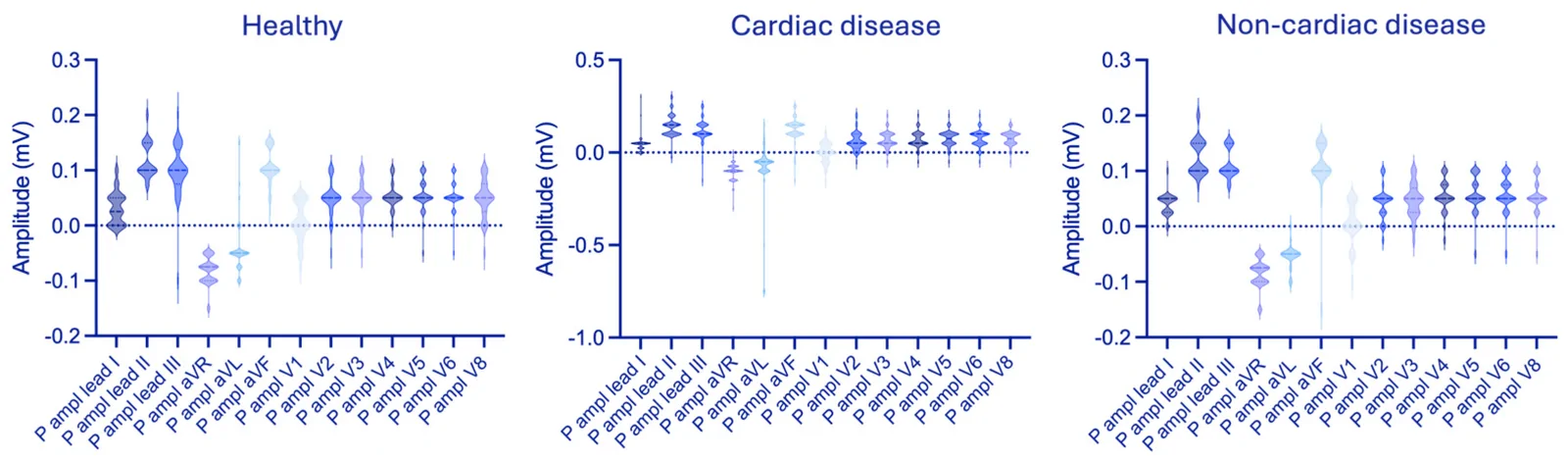
Violin plot with a kernel density estimate representing the distribution of P wave amplitudes across peripheral limb and precordial leads in the healthy, cardiac disease, and non-cardiac disease group. The widest part of a violin plot corresponds to the highest density of values for the given P wave amplitude, with the horizontal dotted line illustrating interquartile range for the given lead. Whiskers (thin lines) represent data points within the 1.5× interquartile range from the quartiles. Small bulges indicate multimodal distribution of P wave amplitude across the lead. ampl = amplitude.

**Table 1:** Thirteen-lead electrocardiography-derived variables from a group of healthy cats and cats with cardiac and non-cardiac disease.

ECG Variable	H (40)					CaD (57)				NCD (28)				Overall *p*-Value
	n	Q1	Median	Q3	Min–Max	n	Q1	Median	Q3	n	Q1	Median	Q3	
P ampl lead I	39	0	0.025	0.05	0–0.1	57	0.025	0.05	0.05	27	0.025	0.05	0.05	0.226
P ampl lead II	40	0.1	0.1	0.15	0.075–0.2	57	0.1	0.15	0.15 *	28	0.1	0.1	0.15	**0.020**
P ampl lead III	40	0.075	0.1	0.138	−0.1–0.2	57	0.1	0.1	0.15	28	0.1	0.1	0.15	0.183
P ampl aVR	40	−0.1	−0.075	−0.075	−0.15–0.05	57	−0.1	−0.1	−0.075 *	28	−0.1	−0.075	−0.075	**0.029**
P ampl aVL	40	−0.069	−0.05	−0.05	−0.1–0.15	57	−0.1	−0.05	−0.05	28	−0.05	−0.05	−0.05	0.441
P ampl aVF	40	0.1	0.1	0.138	0.025–0.15	57	0.1	0.15	0.15 *	28	0.1	0.1	0.15	**0.018**
P ampl V1	39	0	0	0.05	−0.075–0.05	57	−0.037	0	0.05	28	0	0	0.044	0.805
P ampl V2	40	0.05	0.05	0.05	−0.05–0.1	57	0.025	0.05	0.1	28	0.025	0.05	0.05	0.164
P ampl V3	40	0.05	0.05	0.05	−0.05–0.1	57	0.05	0.05	0.1	28	0.025	0.05	0.069 ^#^	**0.014**
P ampl V4	40	0.05	0.05	0.05	0–0.1	57	0.05	0.05	0.1 *	28	0.05	0.05	0.075	**0.020**
P ampl V5	40	0.05	0.05	0.075	−0.05–0.1	57	0.05	0.075	0.1 **	28	0.05	0.05	0.075	**0.006**
P ampl V6	40	0.05	0.05	0.069	−0.05–0.1	57	0.05	0.1	0.1 **	28	0.05	0.05	0.075	**0.002**
P ampl V8	40	0.025	0.05	0.075	−0.05–0.1	57	0.05	0.075	0.1 **	28	0.05	0.05	0.075 ^#^	**<0.001**
P dur lead I	28	30	30	30	0–40	47	30	30	30	25	30	30	30	0.159
P dur lead II	40	30	40	40	30–40	56	30	40	40	28	30	40	40	0.744
P dur lead III	40	30	40	40	30–40	57	30	40	40	28	30	40	40	0.777
P dur aVR	40	30	30	40	30–40	57	30	30	40	28	30	30	40	0.542
P dur aVL	40	30	30	40	30–40	56	30	30	30	28	30	30	30	0.275
P dur aVF	40	30	40	40	30–40	57	30	40	40	28	30	40	40	0.646
P dur V1	33	30	30	40	30–40	52	30	30	40	22	30	30	32.5	0.830
P dur V2	37	30	30	30	30–40	54	30	30	40	24	30	30	40	0.374
P dur V3	37	30	30	30	30–40	55	30	30	40	26	30	30	40	0.305
P dur V4	38	30	30	30	30–40	55	30	30	40	27	30	30	40	0.256
P dur V5	39	30	30	30	30–40	55	30	30	30	28	30	30	40	0.254
P dur V6	39	30	30	30	30–40	55	30	30	30	28	30	30	40	0.435
P dur V8	37	30	30	30	0–40	55	30	30	40	28	30	30	30	0.260
P axis on frontal plane	40	63.44	75.96	90	45–90	55	63.44	71.57	80.54	27	63.44	71.57	80.54	0.449
Q ampl lead I	40	0	0.037	0.05	0–0.1	57	0.013	0.05	0.05	28	0	0.025	0.05	0.153
Q ampl lead II	40	0.025	0.05	0.05	0–0.1	57	0.037	0.05	0.1	28	0.025	0.05	0.05	**0.047**
Q ampl lead III	40	0.006	0.05	0.05	0–0.1	57	0.013	0.05	0.075	28	0	0.05	0.05	0.467
Q ampl aVR	40	0	0	0	0–0.05	57	0	0	0	28	0	0	0	0.833
Q ampl aVL	40	0	0	0.044	0–0.075	56	0	0	0.1 *	28	0	0	0.05	**0.025**
Q ampl aVF	40	0.025	0.05	0.05	0–0.1	57	0.05	0.05	0.075	28	0	0.05	0.05 ^#^	**0.014**
Q ampl V1	40	0	0	0.044	0–0.1	57	0	0	0.05	28	0	0.05	0.05	0.134
Q ampl V2	40	0.006	0.025	0.05	0–0.1	57	0.025	0.05	0.063	28	0.025	0.025	0.05	0.134
Q ampl V3	40	0.025	0.025	0.05	0–0.1	57	0.025	0.05	0.063	28	0.025	0.05	0.05	0.051
Q ampl V4	40	0.025	0.025	0.05	0–0.15	57	0.025	0.05	0.063	28	0	0.037	0.05	0.104
Q ampl V5	40	0.006	0.037	0.05	0–0.2	57	0.037	0.05	0.1 *	28	0.025	0.05	0.05	**0.017**
Q ampl V6	40	0.006	0.025	0.05	0–0.25	57	0.05	0.05	0.1 ***	28	0.025	0.05	0.069	**<0.001**
Q ampl V8	40	0.025	0.05	0.05	0–0.25	57	0.05	0.05	0.1 ***	28	0.013	0.05	0.094	**<0.001**
R ampl lead I	40	0.05	0.05	0.1	0–0.5	57	0.05	0.1	0.175 *	28	0.05	0.1	0.138	**0.034**
R ampl lead II	40	0.1	0.2	0.35	0.025–0.55	57	0.15	0.4	0.5 *	28	0.113	0.25	0.425	**0.028**
R ampl lead III	40	0.1	0.2	0.3	0.05–0.45	57	0.15	0.25	0.45 *	28	0.1	0.175	0.362	**0.019**
R ampl aVR	40	0.025	0.05	0.05	0–0.15	57	0.025	0.05	0.063	28	0.025	0.05	0.1	0.639
R ampl aVL	40	0.025	0.05	0.1	0–0.45	57	0.05	0.1	0.2 *	28	0.05	0.05	0.1	**0.021**
R ampl aVF	40	0.1	0.2	0.35	0.05–0.5	57	0.15	0.35	0.475	28	0.1	0.2	0.375	**0.034**
R ampl V1	40	0.05	0.1	0.138	0.025–0.65	57	0.075	0.1	0.15	28	0.05	0.1	0.2	0.250
R ampl V2	40	0.1	0.125	0.25	0.025–0.75	57	0.1	0.15	0.375	28	0.1	0.2	0.287	0.479
R ampl V3	40	0.1	0.125	0.3	0.025–0.7	57	0.125	0.2	0.4	28	0.1	0.2	0.287	0.120
R ampl V4	40	0.1	0.15	0.287	0.025–0.6	57	0.15	0.25	0.45 *	28	0.1	0.175	0.375	**0.040**
R ampl V5	40	0.1	0.15	0.287	0–0.5	57	0.15	0.3	0.5 **	28	0.1	0.175	0.388	**0.004**
R ampl V6	40	0.056	0.15	0.287	0–0.45	57	0.15	0.3	0.5 **	28	0.1	0.15	0.338	**0.002**
R ampl V8	40	0.1	0.15	0.25	0–0.55	57	0.15	0.25	0.5 **	28	0.081	0.2	0.25 ^#^	**0.001**
S ampl lead I	39	0	0	0.025	0–0.1	57	0	0	0.025	28	0	0	0.025	0.620
S ampl lead II	40	0.025	0.05	0.1	0–0.35	57	0.037	0.05	0.15	28	0.025	0.037	0.237	0.338
S ampl lead III	40	0.025	0.05	0.1	0–0.55	57	0.05	0.1	0.25 *	28	0.025	0.05	0.25	**0.011**
S ampl aVR	39	0.075	0.15	0.25	0–0.4	55	0.05	0.2	0.3	28	0	0.15	0.25	0.176
S ampl aVL	39	0	0.1	0.15	0–0.2	55	0	0.075	0.2	28	0	0	0.138	0.299
S ampl aVF	40	0.025	0.05	0.1	0–0.3	57	0.05	0.1	0.2 *	27	0	0.05	0.25	**0.014**
S ampl V1	40	0.1	0.15	0.2	0.025–0.45	57	0.1	0.2	0.35	28	0.1	0.15	0.375	0.103
S ampl V2	40	0.025	0.05	0.15	0–0.45	57	0.013	0.05	0.15	28	0.025	0.087	0.15	0.770
S ampl V3	40	0	0.05	0.15	0–0.4	57	0.025	0.05	0.1	28	0.006	0.05	0.138	0.985
S ampl V4	40	0	0.037	0.15	0–0.4	57	0.025	0.05	0.087	28	0	0.05	0.1	0.873
S ampl V5	40	0	0.025	0.1	0–0.35	57	0.025	0.05	0.05	28	0	0.025	0.069	0.405
S ampl V6	40	0	0.037	0.1	0–0.25	57	0.025	0.05	0.05	28	0.006	0.025	0.05	0.274
S ampl V8	40	0	0.025	0.05	0–0.1	57	0	0.025	0.05	28	0	0.025	0.05	0.201
V1 R/S ratio	40	0.263	0.67	1	0.08–18	55	0.25	0.5	1	24	0.203	0.67	1	0.607
V2 R/S ratio	31	0.6	1	4	0.13–15	42	0.73	2.75	8	22	0.395	2	5	0.212
V3 R/S ratio	29	0.45	1	5	0.13–14	45	1.5	4	10 *	21	0.44	2	8	**0.023**
V4 R/S ratio	28	0.67	1.33	6.75	0.17–24	44	1.5	5	11	20	0.5	2.5	10.25	0.184
V5 R/S ratio	27	0.8	2	8	0.25–20	45	2.5	6	13	18	1	5	13.5	0.078
V6 R/S ratio	28	1	3	8	0.25–16	43	4	8	12 *	20	4	6	10	**0.039**
V8 R/S ratio	21	1.665	6	10.5	0.5–20	38	3	8.5	14	17	1	6	9	0.223
QRS dur lead I	40	40	40	50	30–60	57	40	50	50	28	40	50	50	0.063
QRS dur lead II	40	50	50	60	40–70	56	50	50	60	28	50	50	60	0.279
QRS dur lead III	40	50	50	60	40–70	57	50	50	60	28	50	50	60	0.128
QRS dur aVR	40	50	50	50	40–70	56	50	50	60 *	28	42.5	50	50	**0.009**
QRS dur aVL	40	40	50	50	40–60	57	50	50	60 **	28	40	50	50 ^##^	**<0.001**
QRS dur aVF	40	50	50	60	40–70	57	50	50	60	28	50	50	60	0.070
QRS dur V1	40	50	50	50	40–70	57	50	50	60	28	50	50	60	0.311
QRS dur V2	40	50	50	50	40–60	57	50	50	60 *	28	50	50	60	**0.035**
QRS dur V3	40	50	50	50	40–60	57	50	50	60	28	50	50	60	0.254
QRS dur V4	40	50	50	50	40–60	57	50	50	60	28	50	50	60	0.061
QRS dur V5	40	50	50	57.5	40–60	57	50	60	60	28	42.5	50	60	0.058
QRS dur V6	40	50	50	50	40–60	57	50	60	60 *	28	50	50	60	**0.016**
QRS dur V8	40	50	50	50	40–70	57	50	60	60 *	28	50	50	60	**0.021**
PQ lead I	26	60	70	80	50–100	47	60	70	80	24	60	70	80	0.763
PQ lead II	40	60	70	70	50–90	56	60	65	70	28	60	70	80	0.342
PQ lead III	40	60	65	70	50–90	57	60	60	70	28	60	70	80	0.321
PQ aVR	40	60	70	80	50–90	56	60	65	70	28	60	70	80	0.271
PQ aVL	40	60	70	70	50–90	56	60	70	77.5	28	60	80	80	0.240
PQ aVF	40	60	70	70	50–90	57	60	60	70	28	60	70	80	0.524
PQ V1	32	52.5	70	80	40–90	52	60	60	70	23	60	70	80	0.454
PQ V2	38	50	70	70	40–80	56	60	60	70	25	60	70	80	0.238
PQ V3	38	50	60	70	40–80	56	60	60	70	27	60	70	80	0.284
PQ V4	38	50	60	70	40–80	56	60	60	70	27	60	70	80	0.249
PQ V5	39	60	60	70	40–80	56	60	60	70	28	60	70	80	0.442
PQ V6	39	60	60	70	40–80	56	60	60	70	28	60	70	80	0.107
PQ V8	37	60	70	70	50–90	56	60	65	70	28	60	65	80	0.757
QRS axis on frontal plane	40	45	51.34	51.34	45–63.44	57	45	50	51.34	28	45	50.19	51.34	0.488
ST lead I	40	0	0	0	0–0	57	0	0	0	28	0	0	0	**0.046**
ST lead II	40	0	0	0.025	0–0.05	57	0	0	0.025	28	0	0	0.05	0.943
ST lead III	40	0	0	0	0–0.05	57	0	0	0.05	28	0	0	0.037	0.435
ST aVR	40	0	0	0	−0.05–0	57	0	0	0	28	0	0	0	0.468
ST aVL	40	0	0	0	−0.05–0	57	0	0	0	28	0	0	0	0.503
ST aVF	40	0	0	0.05	0–0.05	57	0	0	0.05	28	0	0	0.044	0.965
ST V1	40	0	0	0.037	−0.05–0.1	57	0	0	0.05	28	0	0	0.05	0.526
ST V2	40	0	0	0.05	0–0.05	57	0	0	0	28	0	0	0.025	0.482
ST V3	40	0	0	0.019	0–0.05	57	0	0	0	28	0	0	0.025	0.443
ST V4	40	0	0	0	0–0.1	57	0	0	0	28	0	0	0	0.729
ST V5	40	0	0	0	0–0.075	57	0	0	0	28	0	0	0	0.351
ST V6	40	0	0	0	0–0.05	57	0	0	0	28	0	0	0	0.334
ST V8	40	0	0	0	−0.05–0	57	0	0	0	28	0	0	0	0.775
QT lead I	30	160	180	190	150–230	52	160	175	190	19	170	190	200	0.118
QT lead II	40	170	180	200	150–270	55	180	180	200	28	170	190	200	0.413
QT lead III	40	170	180	190	140–270	56	172.5	185	200	26	170	190	210	0.341
QT aVR	40	162.5	180	180	150–260	55	170	180	200	28	170	180	200	0.170
QT aVL	37	160	170	180	150–260	54	170	180	200	25	175	180	200	0.115
QT aVF	40	170	180	197.5	150–260	56	170	185	197.5	28	180	190	200	0.166
QT V1	38	170	180	182.5	150–260	55	170	190	200	27	180	190	200	0.108
QT V2	40	180	180	200	150–260	56	180	190	200	28	180	195	200	0.213
QT V3	40	180	185	190	150–240	56	172.5	180	200	28	180	200	207.5	0.064
QT V4	40	180	180	190	150–260	56	170	190	200	28	180	200	200	0.129
QT V5	40	172.5	185	197.5	150–260	55	170	180	200	27	170	200	200	0.333
QT V6	38	170	180	190	150–240	54	170	180	200	27	180	190	200	0.210
QT V8	36	172.5	180	190	150–270	52	170	180	190	27	180	190	200	0.215
QTc	40	187.9	201.9	211.1	165.2–277.9	55	194.3	205.3	220.2	28	190.7	210.2	223.3	0.243
T lead I	40	0	0	0.025	−0.15–0.1	56	−0.069	−0.037	0 **	28	0	0	0.025 ^##^	**<0.001**
T lead II	40	0.075	0.1	0.15	−0.2–0.25	56	−0.1	0.05	0.1 *	28	0.05	0.1	0.15	**0.022**
T lead III	40	0.056	0.1	0.15	−0.1–0.2	56	−0.05	0.1	0.15	28	0	0.1	0.15	0.342
T aVR	40	−0.1	−0.05	0	−0.2–0.15	56	−0.05	0	0.094 *	28	−0.1	−0.05	−0.031 ^##^	**<0.001**
T aVL	40	−0.1	−0.05	0	−0.75–0.05	56	−0.1	−0.05	0.044	28	−0.075	−0.05	0	0.384
T aVF	40	0.075	0.1	0.15	−0.15–0.25	56	−0.1	0.05	0.1 *	28	0	0.1	0.15	**0.025**
T V1	40	0.05	0.1	0.1	−0.1–0.25	56	0.075	0.1	0.15	28	0.05	0.1	0.15	0.258
T V2	40	0.05	0.1	0.15	−0.15–0.4	56	0	0.1	0.15	28	0.056	0.1	0.15	0.275
T V3	40	0.031	0.1	0.188	−0.15–0.4	56	0	0.1	0.15	28	0.075	0.1	0.15	0.075
T V4	40	0.031	0.1	0.15	−0.2–0.45	56	0	0.05	0.1	28	0.013	0.1	0.15	0.062
T V5	40	0	0.1	0.15	−0.2–0.3	56	−0.05	0.05	0.1 *	28	0	0.087	0.15	**0.027**
T V6	40	0	0.1	0.15	−0.2–0.3	56	−0.087	0.05	0.1 *	28	−0.05	0.063	0.15	**0.043**
T V8	40	0	0.05	0.094	−0.2–0.25	56	−0.1	−0.05	0.037 **	28	−0.05	0	0.087 ^#^	**<0.001**
T axis	40	63.44	90	90	−153.4–180	56	−90	90	116.6	24	65.47	90	90	0.907

Variables with *p* < 0.05 are highlighted in bold. * = *p* < 0.05; ** = *p* < 0.01; *** = *p* < 0.001 between H and CaD; ^#^ = *p* < 0.05; ^##^ = *p* < 0.01. Missing values resulted from the absence of the corresponding wave in some traces. ampl = amplitude; CaD = cardiac disease; dur = duration; ECG = electrocardiogram; H = healthy; n = number; MEA = mean electrical axis; Min–Max = minimum and maximum values; NCD = non-cardiac disease; QTc = corrected QT interval; Q1 = first quartile; Q3 = third quartile.

**Table 2:** Multivariable logistic regression analysis of the selected thirteen-lead electrocardiography-derived variables to discriminate between healthy cats and cats with cardiac disease.

ECG Variable	Wald χ^2^	*p*-Value	Unit of Increase	OR	95% CI
R ampl lead II	13.225	<0.001	0.05 mV	1.508	1.21–1.88
P ampl V8	5.02	0.025	0.05 mV	2.541	1.12–5.74
R ampl aVL	6.218	0.013	0.05 mV	1.536	1.10–2.15
R ampl V6	3.214	0.073	0.05 mV	1.178	0.98–1.41
Age	3.753	0.053	year	1.138	1.00–1.30

ampl = amplitude; CI = confidence interval; OR = odds ratio.

**Table 3:** Pairwise comparison of P wave amplitudes between all 13 electrocardiographic leads in healthy cats (n = 39).

	P ampl I	P ampl II	P ampl III	P ampl aVR	P ampl aVL	P ampl aVF	P ampl V1	P ampl V2	P ampl V3	P ampl V4	P ampl V5	P ampl V6	P ampl V8
P ampl I	1	**<0.001**	**<0.001**	**<0.001**	0.057	**<0.001**	0.904	0.959	0.861	0.689	0.398	0.617	0.868
P ampl II	**<0.001**	1	0.987	**<0.001**	**<0.001**	1	**<0.001**	**<0.001**	**<0.001**	**<0.001**	**<0.001**	**<0.001**	**<0.001**
P ampl III	**<0.001**	0.987	1	**<0.001**	**<0.001**	1	**<0.001**	**<0.001**	**0.001**	**0.003**	**0.014**	**0.005**	**0.001**
P ampl aVR	**<0.001**	**<0.001**	**<0.001**	1	0.949	**<0.001**	0.087	**<0.001**	**<0.001**	**<0.001**	**<0.001**	**<0.001**	**<0.001**
P ampl aVL	0.057	**<0.001**	**<0.001**	0.949	1	**<0.001**	0.919	**<0.001**	**<0.001**	**<0.001**	**<0.001**	**<0.001**	**<0.001**
P ampl aVF	**<0.001**	1	1	**<0.001**	**<0.001**	1	**<0.001**	**<0.001**	**<0.001**	**<0.001**	**0.002**	**0.001**	**<0.001**
P ampl V1	0.904	**<0.001**	**<0.001**	0.087	0.919	**<0.001**	1	0.087	**0.037**	**0.014**	**0.003**	**0.010**	**0.039**
P ampl V2	0.959	**<0.001**	**<0.001**	**<0.001**	**<0.001**	**<0.001**	0.087	1	1	1	1	1	1
P ampl V3	0.861	**<0.001**	**0.001**	**<0.001**	**<0.001**	**<0.001**	**0.037**	1	1	1	1	1	1
P ampl V4	0.689	**<0.001**	**0.003**	**<0.001**	**<0.001**	**<0.001**	**0.014**	1	1	1	1	1	1
P ampl V5	0.398	**<0.001**	**0.014**	**<0.001**	**<0.001**	**0.002**	**0.003**	0.999	1	1	1	1	1
P ampl V6	0.617	**<0.001**	**0.005**	**<0.001**	**<0.001**	**0.001**	**0.010**	1	1	1	1	1	1
P ampl V8	0.868	**<0.001**	**0.001**	**<0.001**	**<0.001**	**<0.001**	**0.039**	1	1	1	1	1	1

Variables with a *p* < 0.05 are highlighted in bold. ampl = amplitude.

**Table 4:** Pairwise comparison of P wave amplitudes between all 13 electrocardiographic leads in cats with cardiac disease (n = 57).

	P ampl I	P ampl II	P ampl III	P ampl aVR	P ampl aVL	P ampl aVF	P ampl V1	P ampl V2	P ampl V3	P ampl V4	P ampl V5	P ampl V6	P ampl V8
P ampl I	1	**<0.001**	**<0.001**	**<0.001**	**0.006**	**<0.001**	0.661	0.969	0.311	0.158	**0.016**	**0.016**	**0.026**
P ampl II	**<0.001**	1	0.719	**<0.001**	**<0.001**	0.986	**<0.001**	**<0.001**	**<0.001**	**<0.001**	**<0.001**	**<0.001**	**<0.001**
P ampl III	**<0.001**	0.719	1	**<0.001**	**<0.001**	1	**<0.001**	**<0.001**	**0.006**	**0.018**	0.172	0.172	0.121
P ampl aVR	**<0.001**	**<0.001**	**<0.001**	1	0.964	**<0.001**	0.052	**<0.001**	**<0.001**	**<0.001**	**<0.001**	**<0.001**	**<0.001**
P ampl aVL	**0.006**	**<0.001**	**<0.001**	0.964	1	**<0.001**	0.808	**<0.001**	**<0.001**	**<0.001**	**<0.001**	**<0.001**	**<0.001**
P ampl aVF	**<0.001**	0.986	1	**<0.001**	**<0.001**	1	**<0.001**	**<0.001**	**<0.001**	**0.001**	**0.022**	**0.022**	**0.013**
P ampl V1	0.661	**<0.001**	**<0.001**	0.052	0.808	**<0.001**	1	**0.028**	**<0.001**	**<0.001**	**<0.001**	**<0.001**	**<0.001**
P ampl V2	0.969	**<0.001**	**<0.001**	**<0.001**	**<0.001**	**<0.001**	**0.028**	1	0.994	0.957	0.539	0.539	0.644
P ampl V3	0.311	**<0.001**	**0.006**	**<0.001**	**<0.001**	**<0.001**	**<0.001**	0.994	1	1	0.997	0.997	0.999
P ampl V4	0.158	**<0.001**	**0.018**	**<0.001**	**<0.001**	**0.001**	**<0.001**	0.957	1	1	1	1	1
P ampl V5	**0.016**	**<0.001**	0.172	**<0.001**	**<0.001**	**0.022**	**<0.001**	0.539	0.997	1	1	1	1
P ampl V6	**0.016**	**<0.001**	0.172	**<0.001**	**<0.001**	**0.022**	**<0.001**	0.539	0.997	1	1	1	1
P ampl V8	**0.026**	**<0.001**	0.121	**<0.001**	**<0.001**	**0.013**	**<0.001**	0.644	0.999	1	1	1	1

Variables with a *p* < 0.05 are highlighted in bold. ampl = amplitude.

**Table 5:** Pairwise comparison of P wave amplitudes between all 13 electrocardiographic leads in cats with non-cardiac disease (n = 27).

	P ampl I	P ampl II	P ampl III	P ampl aVR	P ampl aVL	P ampl aVF	P ampl V1	P ampl V2	P ampl V3	P ampl V4	P ampl V5	P ampl V6	P ampl V8
P ampl I	1	**<0.001**	**<0.001**	**0.001**	**0.016**	**<0.001**	0.830	1	1	0.991	0.926	0.895	0.979
P ampl II	**<0.001**	1	1	**<0.001**	**<0.001**	1	**<0.001**	**<0.001**	**<0.001**	**0.001**	**0.003**	**0.004**	**0.001**
P ampl III	**<0.001**	1	1	**<0.001**	**<0.001**	1	**<0.001**	**<0.001**	**<0.001**	**0.005**	**0.021**	**0.028**	**0.009**
P ampl aVR	**0.001**	**<0.001**	**<0.001**	1	1	**<0.001**	0.264	**<0.001**	**<0.001**	**<0.001**	**<0.001**	**<0.001**	**<0.001**
P ampl aVL	**0.016**	**<0.001**	**<0.001**	1	1	**<0.001**	0.811	**0.009**	**0.002**	**<0.001**	**<0.001**	**<0.001**	**<0.001**
P ampl aVF	**<0.001**	1	1	**<0.001**	**<0.001**	1	**<0.001**	**<0.001**	**0.001**	**0.012**	**0.044**	0.057	**0.020**
P ampl V1	0.830	**<0.001**	**<0.001**	0.264	0.811	**<0.001**	1	0.726	0.477	0.112	**0.037**	**0.028**	0.077
P ampl V2	1	**<0.001**	**<0.001**	**<0.001**	**0.009**	**<0.001**	0.726	1	1	0.998	0.968	0.950	0.993
P ampl V3	1	**<0.001**	**<0.001**	**<0.001**	**0.002**	**0.001**	0.477	1	1	1	0.997	0.994	1
P ampl V4	0.991	**0.001**	**0.005**	**<0.001**	**<0.001**	**0.012**	0.112	0.998	1	1	1	1	1
P ampl V5	0.926	**0.003**	**0.021**	**<0.001**	**<0.001**	**0.044**	**0.037**	0.968	0.994	1	1	1	1
P ampl V6	0.895	**0.004**	**0.028**	**<0.001**	**<0.001**	0.057	**0.028**	0.950	0.994	1	1	1	1
P ampl V8	0.979	**0.001**	**0.009**	**<0.001**	**<0.001**	**0.020**	0.077	0.993	1	1	1	1	1

Variables with a *p* < 0.05 are highlighted in bold. ampl = amplitude.

## Data Availability

The data presented in this study are available on request from the corresponding author.

## Abbreviations

The following abbreviations are used in this manuscript:

AUC	Area under the curve
CaD	Cardiac disease
CI	Confidence interval
ECG	Electrocardiogram
H	Healthy
HCM	Hypertrophic cardiomyopathy
IQR	Interquartile range
NCD	Non-cardiac disease
OR	Odds ratio
VIF	Variance inflation factor

## Appendix A

**Table A1:** Echocardiographic variables obtained from all cats in each group.

Variable	H (40)			CaD (57)			NCD (28)			Overall
	Q1	Median	Q3	Q1	Median	Q3	Q1	Median	Q3	*p*-Value
LAD (mm)	13.47	14.45	16.18	15.32	17.1	19.45 ***	13.93	15.15	16.88 ^##^	**<0.001**
RAD (mm)	10.13	11.64	12.8	9.69	10.77	12.05	10.38	11.27	12.74	0.280
LA/Ao	1.33	1.43	1.5	1.5	1.6	1.85 ***	1.39	1.49	1.58 ^##^	**<0.001**
RVFWd (mm)	2.09	2.41	2.6	2.31	2.59	2.95	2.1	2.425	2.7	0.013
RVIDd (mm)	6.87	7.69	8.88	6.38	7.33	8.1	6.9	7.555	8.69	0.292
LVWT max (mm)	3.7	4.05	4.44	4.97	5.66	6.45 ***	3.92	4.245	5.11 ^###^	**<0.001**
LVIDd (mm)	13.58	14.95	16.24	13.41	14.71	16	13.9	15.75	16.83	0.336
LVIDs (mm)	5.62	7.42	8.94	5.36	6.29	8.13	5.73	7.42	9.1	0.153
LV FS (%)	43.5	51	58	47.5	53	60	45	50	57	0.299
IVSd (mm)	3.49	3.73	4.24	4.54	5	5.75 ***	3.4	3.8	4.17 ^###^	**<0.001**
LVFWd (mm)	3.6	3.9	4.32	4.71	5.35	6.24 ***	3.7	4.09	5.03 ^###^	**<0.001**
LVOT Vmax (m/s)	0.9	1	1.1	1	1.6	3 ***	0.8	0.95	1.1 ^###^	**<0.001**

Variables with *p* < 0.05 are highlighted in bold. *** = *p* < 0.001 between H and CaD; ^##^ = *p* < 0.01, ^###^ = *p* < 0.001 between CaD and NCD. CaD = cardiac disease; H = healthy; IVSd = interventricular septum thickness at end-diastole; LAD/RAD = left/right atrial end-systolic maximal internal diameter in long axis; LA/Ao = end-systolic left atrial diameter and aortic root diameter ratio; LV FS = left ventricular fractional shortening; LVIDd/s = left ventricular internal diameter at end-diastole/end-systole; LVOT Vmax = left ventricular outflow tract maximal velocity; LVWT max = maximal left ventricular free wall thickness at end-diastole in long- or short-axis view; LVFWd = left ventricular free wall thickness at end-diastole; NCD = non-cardiac disease; Q1 = first quartile; Q3 = third quartile; RVFWd = right ventricular free wall thickness at end-diastole; RVIDd = right ventricular internal diameter at end-diastole; SAM = systolic anterior motion of mitral valve.

**Table A2:** Adjusted *p* values using the Benjamini–Hochberg FDR procedure.

Variable	Adjusted *p*-Values
P ampl lead I	0.4253
P ampl lead II	0.1196
P ampl lead III	0.3823
P ampl aVR	0.1266
P ampl aVL	0.5628
P ampl aVF	0.1196
P ampl V1	0.8470
P ampl V2	0.3660
P ampl V3	0.1182
P ampl V4	0.1196
P ampl V5	0.0738
P ampl V6	**0.0351**
P ampl V8	**0.0139**
P dur lead I	0.3623
P dur lead II	0.8194
P dur lead III	0.8238
P dur aVR	0.6268
P dur aVL	0.4353
P dur aVF	0.7230
P dur V1	0.8639
P dur V2	0.5118
P dur V3	0.4628
P dur V4	0.4320
P dur V5	0.4320
P dur V6	0.5628
P dur V8	0.4320
P axis on frontal plane	0.5650
Q ampl lead I	0.3535
Q ampl lead II	0.1641
Q ampl lead III	0.5736
Q ampl aVR	0.8639
Q ampl aVL	0.1230
Q ampl aVF	0.1182
Q ampl V1	0.3153
Q ampl V2	0.3153
Q ampl V3	0.1767
Q ampl V4	0.2864
Q ampl V5	0.1196
Q ampl V6	**0.0174**
Q ampl V8	**0.0149**
R ampl lead I	0.1411
R ampl lead II	0.1266
R ampl lead III	0.1196
R ampl aVR	0.7205
R ampl aVL	0.1196
R ampl aVF	0.1411
R ampl V1	0.4320
R ampl V2	0.5812
R ampl V3	0.3029
R ampl V4	0.1520
R ampl V5	**0.0463**
R ampl V6	**0.0351**
R ampl V8	**0.0177**
S ampl lead I	0.7052
S ampl lead II	0.4772
S ampl lead III	0.1154
S ampl aVR	0.3756
S ampl aVL	0.4584
S ampl aVF	0.1182
S ampl V1	0.2864
S ampl V2	0.8238
S ampl V3	0.9854
S ampl V4	0.8982
S ampl V5	0.5434
S ampl V6	0.4353
S ampl V8	0.4114
V1 R/S ratio	0.6960
V2 R/S ratio	0.4143
V3 R/S ratio	0.1211
V4 R/S ratio	0.3823
V5 R/S ratio	0.2250
V6 R/S ration	0.1520
V8 R/S ratio	0.4253
QRS dur lead I	0.1954
QRS dur lead II	0.4371
QRS dur lead III	0.3139
QRS dur aVR	0.0928
QRS dur aVL	**0.0107**
QRS dur aVF	0.2096
QRS dur V1	0.4669
QRS dur V2	0.1411
QRS dur V3	0.4320
QRS dur V4	0.1954
QRS dur V5	0.1937
QRS dur V6	0.1196
QRS dur V8	0.1196
PQ Lead I	0.8238
PQ Lead II	0.4772
PQ Lead III	0.4770
PQ aVR	0.4353
PQ aVL	0.4320
PQ aVF	0.6124
PQ V1	0.5670
PQ V2	0.4320
PQ V3	0.4400
PQ V4	0.4320
PQ V5	0.5628
PQ V6	0.2885
PQ V8	0.8238
QRS axis	0.5826
ST Lead I	0.1641
ST Lead II	0.9568
ST Lead III	0.5628
ST aVR	0.5736
ST aVL	0.5964
ST aVF	0.9724
ST V1	0.6124
ST V2	0.5812
ST V3	0.5628
ST V4	0.8093
ST V5	0.4849
ST V6	0.4772
ST V8	0.8238
QT lead I	0.3029
QT Lead II	0.5493
QT Lead III	0.4772
QT aVR	0.3686
QT aVL	0.2997
QT aVF	0.3660
QT V1	0.2885
QT V2	0.4143
QT V3	0.1954
QT V4	0.3139
QT V5	0.4772
QT V6	0.4143
QT V8	0.4143
QTc	0.4320
T lead I	**0.0107**
T lead II	0.1211
T lead III	0.4772
T aVR	**0.0139**
T aVL	0.5210
T aVF	0.1230
T V1	0.4320
T V2	0.4353
T V3	0.2197
T V4	0.1954
T V5	0.1266
T V6	0.1580
T V8	**0.0174**
T axis	0.9266

Variables with a *p* < 0.05 are highlighted in bold. ampl = amplitude; dur = duration; FDR = false discovery rate.

**Table A3:** Sensitivity analysis: multivariable logistic regression analysis of selected thirteen-lead electrocardiography-derived variables to discriminate between 39 healthy cats and 33 cats with hypertrophic cardiomyopathy phenotype.

ECG Variable	Wald χ^2^	*p*-Value	Unit of Increase	OR	95% CI
R ampl V8	0.355	0.551	0.05 mV	1.109	0.79–1.56
R ampl II	12.543	<0.001	0.05 mV	1.912	1.34–2.74
R ampl V6	3.41	0.065	0.05 mV	1.318	1.00–1.77
R ampl aVL	8.717	0.003	0.05 mV	1.997	1.26–3.16
P ampl V8	6.313	0.012	0.05 mV	5.439	1.45–20.39

ampl = amplitude; CI = confidence interval; OR = odds ratio.

**Table A4:** R/S ratio calculated from the precordial leads in all groups.

Variable	H (n)	CaD (n)	NCD (n)
R/S in lead V1 = 1	9	4	4
R/S in lead V1 > 1	8	13	5
R/S in lead V1 < 1	23	38	15
R/S in lead V2 = 1	7	4	0
R/S in lead V2 > 1	13	27	13
R/S in lead V2 < 1	11	11	9
R/S in lead V3 = 1	4	2	1
R/S in lead V3 > 1	14	35	12
R/S in lead V3 < 1	11	8	8
R/S in lead V4 = 1	2	0	0
R/S in lead V4 > 1	15	35	13
R/S in lead V4 < 1	11	9	7
R/S in lead V5 = 1	4	0	2
R/S in lead V5 > 1	16	36	13
R/S in lead V5 < 1	7	9	3
R/S in lead V6 = 1	5	1	2
R/S in lead V6 > 1	17	36	16
R/S in lead V6 < 1	6	6	2
R/S in lead V8 = 1	3	2	3
R/S in lead V8 > 1	17	34	12
R/S in lead V8 < 1	1	2	2

CaD = cardiac disease; H = healthy; NCD = non-cardiac disease.
